# Emerging healthcare issues and needs of young MSM & TG in India: needs assessment from multi-stakeholders perspective

**DOI:** 10.1186/1471-2334-12-S1-P91

**Published:** 2012-05-04

**Authors:** Maheswar Satpathy

**Affiliations:** 1 National Centre in HIV Social Research, University of New South Wales (UNSW), Sydney, Australia

## Background

There is inadequate research in India on young MSM and TGs and not much multi-systemic insight is taken into healthcare decisions. Hence, healthcare needs and issues of young MSM & TGs need to be studied from a multi-stakeholder perspective.

## Methods

An online pilot survey containing a self-administered 14 item questionnaire exploring perception of problems, needs and associated factors was conducted. 52 participants took part in this survey. Data was analyzed using simple statistics, and qualitative responses were thematically analyzed using QDA.

## Results

a) Problems: young MSMs & TGs engage in unprotected anal intercourse (80.9%), risk sex with people unknown of their HIV status (66%), drug and tobacco (59.6%) and alcohol consumption (55.3%). All participants perceived that MSM & TGs don’t use condoms consistently. They also experience mental health problems like anxiety and depression (80.9%).

b) Associated factors: QDA outlined following major factors for engagement with health risk behaviours: i) criminalization of homosexuality; ii) social alienation, lack of social/familial support and poor acceptance; iii) psychological trauma, loneliness; iv) lack of knowledge about safe sex & STDs; v) problems forming a coherent self-identity; vi) fear of losing partner and inability to maintain stable relationship; vii) condoms as barriers in pleasure and engagement for thrill and viii) poor self-esteem and peer-pressure; ix) financial needs.

## Conclusion

Healthcare needs and issues of young MSM & TG needs to be emphasized with more comprehensive understanding of their social positioning and psycho-social needs than a mere emphasis on HIV/AIDS.

